# Half Metallic Ferromagnetism and Transport Properties of Zinc Chalcogenides ZnX_2_Se_4_ (X = Ti, V, Cr) for Spintronic Applications

**DOI:** 10.3390/ma15010055

**Published:** 2021-12-22

**Authors:** Mohsen Al-Qhtani, Ghulam M. Mustafa, Nasheeta Mazhar, Sonia Bouzgarrou, Qasim Mahmood, Abeer Mera, Zaki I. Zaki, Nasser Y. Mostafa, Saad H. Alotaibi, Mohammed A. Amin

**Affiliations:** 1 Materials Science and Engineering Group, Department of Chemistry, Faculty of Science, Taif University, Taif 21944, Saudi Arabia; mohsen@tu.edu.sa (M.A.-Q.); zakimohamed2000@yahoo.com (Z.I.Z.); nmost69@yahoo.com (N.Y.M.); s.alosaimi@tu.edu.sa (S.H.A.); Mohamed@tu.edu.sa (M.A.A.); 2 Department of Physics, Division of Science & Technology, University of Education, Lahore 54000, Pakistan; 3 Department of Physics, University of Lahore, Lahore 05422, Pakistan; nasheen@gmail.com; 4 Laboratoire de Microélectronique et Instrumentation (UR 03/13–04), Faculté des Sciences de Monastir, Avenue de l’Environnement, Monastir 5000, Tunisia; b_sona3@yahoo.fr; 5 Department of Physics, College of Science, Qassim University, P.O. Box 64, Buraydah 51452, Saudi Arabia; 6 Basic and Applied Scientific Research Center, Imam Abdulrahman Bin Faisal University, P.O. Box 1982, Dammam 31441, Saudi Arabia; 7 Department of Physics, College of Science, Imam Abdulrahman Bin Faisal University, P.O. Box 1982, Dammam 31441, Saudi Arabia; 8 Department of Physics, College of Arts and Science, Prince Sattam Bin Abdulaziz University, Wadi Addawasir 11991, Saudi Arabia; abeer.mera047@gmail.com; 9 Department of Physics, Faculty of Science, Kafrelsheikh University, Kafrelsheikh 33516, Egypt

**Keywords:** Zn based chalcogenides, density functional theory, half metallic ferromagnetism, transport properties

## Abstract

In ferromagnetic semiconductors, the coupling of magnetic ordering with semiconductor character accelerates the quantum computing. The structural stability, Curie temperature (T_c_), spin polarization, half magnetic ferromagnetism and transport properties of ZnX_2_Se_4_ (X = Ti, V, Cr) chalcogenides for spintronic and thermoelectric applications are studied here by density functional theory (DFT). The highest value of T_c_ is perceived for ZnCr_2_Se_4_. The band structures in both spin channels confirmed half metallic ferromagnetic behavior, which is approved by integer magnetic moments (2, 3, 4) μ_B_ of Ti, V and Cr based spinels. The HM behavior is further measured by computing crystal field energy ΔE _crystal_, exchange energies Δ _x_(*d*), Δ _x_ (*pd*) and exchange constants (N_o_ α and N_o_ β ). The thermoelectric properties are addressed in terms of electrical conductivity, thermal conductivity, Seebeck coefficient and power factor in within a temperature range 0–400 K. The positive Seebeck coefficient shows p-type character and the PF is highest for ZnTi2Se4 (1.2 × 10^11^ W/mK^2^) among studied compounds.

## 1. Introduction

The continuous increasing demand of data storage devices in the current scenario has triggered the scientific community to exploit materials in having additional degrees of freedom. Therefore, advanced technology demands highly modern devices with reduce sizes and better efficiency. A few decades ago, the coupling of spin of electrons with its charge laid the basis for new debate among scientists and engineers across the globe, giving rise to an emerging field of spintronics [1,2]. This concept modernized quantum computing based on fast speed computers by calculating the spin current in spin up and spin down channels separately instead of charge in conventional electronics. Therefore, it accelerates the data processing speed and enhances the data storage capability of spintronic devices. This effect of combining of spin with charge of electrons led to the reduction in size, improvement in efficiency and enhanced the non-volatility of solid-state devices [3,4,5].

The developing concept of half metallic ferromagnetism reorganized the spintronic industry and performance, playing a critical role in devices such as magneto-resistive random-access memory (MRAM), quantum computing, read head of magnetic hard drive, magnetic sensors, spin valves and giant magneto-resistive effect (GMR) [6,7]. Half metallic ferromagnets (HMF) show a conducting behavior in one spin channel while semiconducting or insulating in other. Therefore, due to the presence of gap at fermi level in one spin channel, the density of states at Fermi level must be 100% spin polarized. Initially, the concept of HMF was launched by de-Groot et al. [8,9] in half-Heusler alloys. Later, it was explored experimentally as well as theoretically in a variety of materials. Theoretically, HMF was examined in pure and doped transition metals, double perovskites and Heusler alloys [10,11,12]. The Zn based spinel chalcogenides are incipient as a new candidate for spintronic because of their high temperature half metallic ferromagnetic (HMF) behavior.

The main challenges in the fabrication of spintronic devices include the existence of ferromagnetism at low temperature and phase instability at high temperature (RT). According to previous investigations, the origin of half metallic ferromagnetism is still unclear i.e., either it occurs due to spin of electrons or due to accumulation of the magnetic ions [13,14]. Previously, many reports claimed that half metallic ferromagnetism originates from grouping of magnetic ions and secondary phases. To resolve this issue, much work is carried out by doping the non-magnetic elements into the non-magnetic alloys that improved our understanding regarding quantum mechanical states in real materials, which enhance the data storage capability of the spintronic devices. Since the digital information is recorded in terms of binary states of 0 and 1, which represents the spin up and spin down states here. In one spin channel there exists a gap at fermi level while for other spin channels, it behaves like conductors. [15,16].

In the recent past, extensive research has been carried out on optoelectronic, magnetic and thermoelectric properties of spinel chalcogenides. The single crystal of ZnFe_2_S_4_ has been grown experimentally for electronic and optical properties which has promising applications [17]. The spinels XCr_2_Z_4_ (X = Zn, Cd, Hg; Z = S, Se) show different phases at variant pressures. The careful analysis of XRD, and Raman spectroscopic on ZnCr_2_S_4_ confirm the two structural transitions at 22 GPa (I41/amd phase) and 33 GPa (orthorhombic phase). In addition, a thorough investigation of the Fd3m phase ensures their existence at ambient conditions [18]. The nanoparticles of ZnCr_2_S_4_ are fabricated by mechanical alloying method and investigated the magnetism with effective magnetic moment m_eff_ = 2.089 mB/f.u and short-range magnetic interaction [19]. The single crystal Ta-doped ZnCr_2_Se_4_ are prepared by vapor transport method to study the impact on thermodynamic, magnetic, and structural properties. The small doping of Ta changes the direct magnetic interaction and transport behavior [20]. The theoretical study on these has not been done. Therefore, we have explored the ZnX_2_Se_4_ (X = Ti, V, Cr) theoretically, which provides evidence of the researcher’s realization of applications of these compounds. The aim of study is the reporting of room temperature ferromagnetism due to exchange mechanism of electron spin rather than clustering effects of magnetic elements and effect of transport parameters on spintronic devices. The findings of integer magnetic moment and complete spin polarization increases their importance for spintronics. Moreover, a high value of power factor is found for ZnV_2_Se_4_.

## 2. Method of Calculations

DFT calculations provide an efficient approach to compute the properties of the materials before realizing their applications for experimental analysis. The DFT has proven to be one of the most accurate theories for the calculation of the electronic structure of solids [21]. Here, WEIN2K software, which works according to full potential linearized augmented plane wave (FP-LAPW) is incorporated for calculations [13,14]. The optimization of energy of electrons involves the calculation of eigen value as well as eigen function of Kohn–Sham expressions. To probe the ground state potential and mechanical properties, the Generalized Gradient Approximation (GGA) was employed. The calculation of band gap was performed based on modified Becke–Johnson functional of Koller et al. 2012 [22] which give the value of band gap close to the experimental value. The self-consistent converged values of c are calculated by:

(1) $$V_{x1\sigma }^{mBJ}\left(r\right)=cV_{x1\sigma }^{BR}\left(r\right)+(3c-2)\;\frac{1}{\pi }\frac{\sqrt{5}}{12}\frac{\sqrt{2t_{\sigma }\left(r\right)}}{\rho_{\sigma }\left(r\right)}$$

where density of the state is shown by $\rho_{\sigma }\left(r\right)$ , K.E density is shown by $t_{\sigma }\left(r\right)$ and $V_{x1\sigma }^{BR}\left(r\right)$ is Becke Roussel potential (V*^BR^*) and C can be calculated as:

(2) $$c=\alpha +(\beta \frac{1}{Vcell}ʃd^{3}r\;(\frac{\left|\nabla \rho \left(r\right)\right|}{\rho \left(r\right)})$$

where α and β behaves as free parameters into WEIN2K code, α = −0.012 and β = 1.023 Bohr^1/2^.

To avoid the overlapping of core with the interstitial regions, the core region is described by the product of radial and harmonic function while interstitial region is described by the plane wave basis. Here we treated the valence states as scalar relativistic while the core region was fully relativistic. Inside the interstitial region, the cut of point of plane wave was adjusted by setting the value of *R_MT_ × K_max_*. In present case the value of this product is set 8. Here, *R_MT_* represents the smallest value of muffin-tin radius. Inside the sphere, the expansion of spherical harmonic is controlled by the value of angular momentum *ι_max_* (10) while charge expansion is controlled by the value of *G_max_* (18). The SCF was run until the difference between two successive measurements became less than 10^−2^ mRy. For the computation of thermoelectric properties, we need a dense mesh; therefore, we increased the number of k-points to 2000. Finally, thermoelectric parameters were computed using BoltZTraP code based on semi-classical Boltzmann equations [23].

## 3. Results and Discussion

### 3.1. Structure Behavior and Thermodynamically Stability

**Figure 1:**
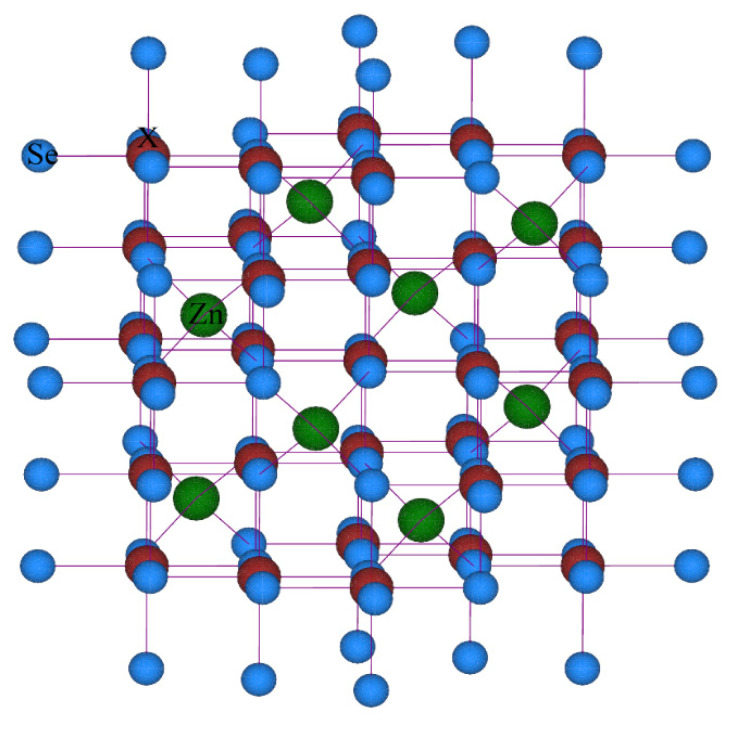
The crystal structure of ZnX_2_Se_4_ (X = Ti, V, Cr) formed by VESTA Software.

The structure of cubic unit cell of spinel chalcogenides ZnX_2_Se_4_ (X = Ti, V, Cr) constructed using VESTA software is shown in Figure 1. This unit cell was optimized in WIEN2K software by giving information on space group (*Fd3m*) and atomic position. The observed lattice constant (a_o_), Bulk modulus (B_o_)*,* Cohesive energy (*E_coh_*), Formation energy, (Δ*H_f_*) and Curie Temperature T_c_ (K) for these compositions ZnX_2_Se_4_ (X = Ti, V, Cr) are presented in Table 1. The lattice constant decreases from Ti to V and Cr. This decrease in value of lattice constant is attributed to the decreases of ionic radii of Ti (0.67Å), V (0.64Å) and Cr (0.61Å) [19].

**Figure 2:**
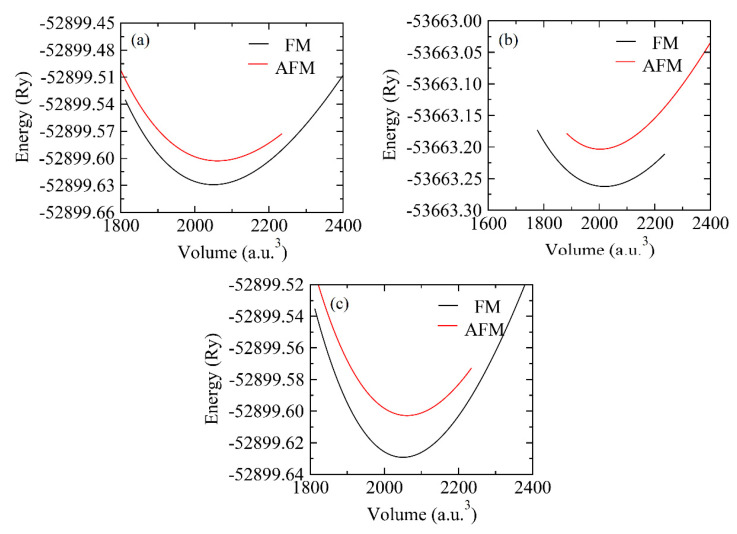
The optimization plots of (**a**) ZnTi_2_Se_4_, (**b**) ZnV_2_Se_4_, (**c**) ZnCr_2_Se_4_ in antiferromagnetic (AFM) and ferromagnetic (FM) phases.

Stiffness of any material is one of the important factors, which determines its mechanical strength. The stiffness of the material is measured in terms of Bulk modulus (B_o_) when Ti is substituted with V and Cr (See Table 1). This increase in the value of B_o_ is credited to the increase in number of *d*-shell electrons from Ti to Cr. To ensure, in which phase compounds are more stable, their energy volume curves are plotted in ferromagnetic and antiferromagnetic phases separately and positive value of energy difference (∆E = E_AFM_ − E_FM_) indicated that these compositions are more stable in the FM state than in the AFM state as shown in Figure 2. To further ensure the stability of the FM state, the formation energy is calculated by the following expression:

(3) $$\Delta H_{f}=E_{Total}\left(Zn_{l}\;Se_{n}\right)-lE_{Zn}-mE_{X}-nE_{Se}$$

where $E_{Total}\left(Zn_{l}X_{m}Se_{n}\right)$ is the total energy of the ZnX_2_Se_4_ (X = Ti, V, Cr) chalcogenides, *E_Zn_*, *E_X_* and the *E_Se_* gives energies of individual elements. The negative value of this formation energy assures the stability of these materials (See Table 1). It is evident from formation energy that Ti based composition is most stable. Cohesive energy also measures the stability of materials which is computed by relation

(4) $$∆E_{coh}\;=\;aE_{X}^{atom}+bE_{Zn}^{atom}+cE_{Se}^{atom}-E_{Total}(\mathrm{Zn},\;X,\;\mathrm{Se})$$

Here *E_Total_* = (Zn_a_ X_b_ Se_c_) is the total energy of compounds and $E_{X}^{Atom}$ , $\;E_{Zn}^{Atom}$ and $E_{Se}^{Atom}$ are energies of individual atoms. The cohesive energies of ternary compounds are larger than their binary counterparts, which shows the compounds are thermodynamically stable [25].

The curie temperature (T_c_) is the important factor which disturb experimental fabrication for storage devices. The T_c_ is by formula, T_c_ = 2 Δ *E*/3Xk_B._ Where k_B_ and X are Boltzmann constant and the content of doping elements (X = Ti, V, Cr), respectively [26]. The calculated values of T_c_ for ZnX_2_Se_4_ are shown in Table 1. The ZnCr_2_Se_4_ has room temperature ferromagnetism.

The higher value of T_c_ for Cr (3.88µ_B_) is due to its higher magnetic moment than V (2.83µ_B_) and Ti (1.73µ_B_). Therefore, the above prediction of RT ferromagnetism makes these alloys a potential applicant for spintronics.

### 3.2. Band Structure and Density of States

**Figure 3:**
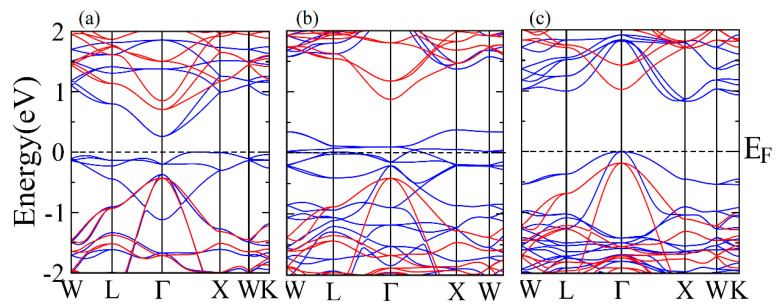
The band structures for up -spin channel (blue color) and down- spin channel (red color) of (**a**) ZnTi_2_Se_4_, (**b**) ZnV_2_Se_4_ and (**c**) ZnCr_2_Se_4_.

The spin polarization is calculated from the band structure of ZnX_2_Se_4_ (X = Ti, V, Cr) at the equilibrium lattice constants along different symmetry directions in first Brillion zone. The compounds in spin-up configuration reveal that the valence band maxima and conduction band minima lie at different symmetry points of the Brillion zone and some of the states of valence band cross the fermi level which exposed the metallic nature in this configuration. In addition, when band structure in spin-down channel is plotted it exhibited the direct bandgap semiconducting nature where valence band maxima and conduction band minima lie at Γ-symmetry point (See Figure 3a–c). In spin down channel, the energy states lie far from fermi level, which is attributed to the strong *p*-*d* splitting. The ferromagnetic semiconductors have the ability to combine magnetic and semiconducting characteristics in a single device because of this splitting. Therefore, replacement of transition metals in octahedra environment of spinel chalcogenides imparts the multifunctional characteristics to these materials.

**Figure 4:**
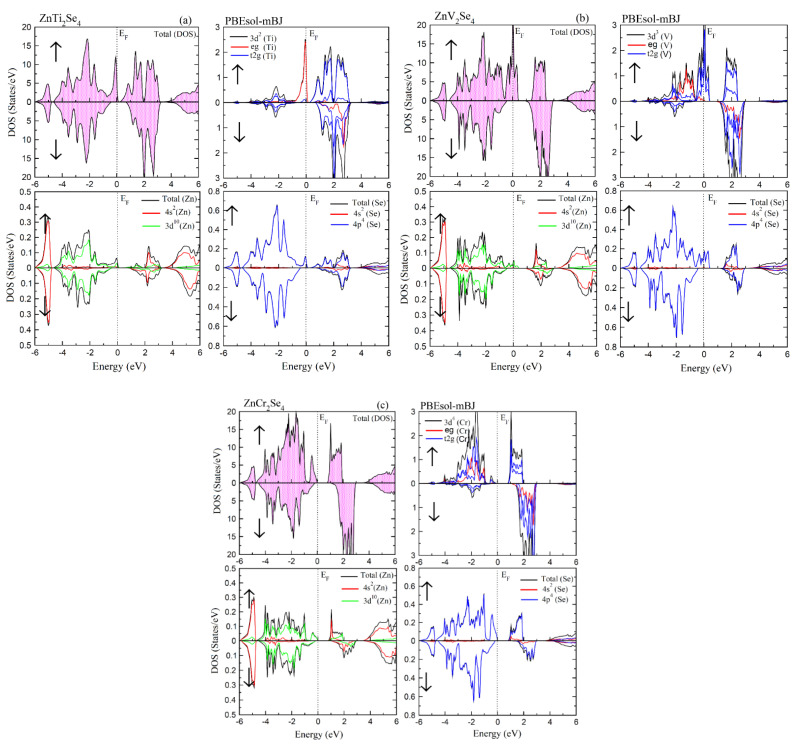
(**a**) The total DOS of ZnTi_2_Se_4_ and PDOS of Zn, Ti, S formed by PBEsol-mBJ; (**b**) the total DOS of ZnV_2_Se_4_ and PDOS of Zn, V, S formed by PBEsol-mBJ; And (**c**) the total DOS of ZnCr_2_Se_4_ and PDOS of Zn, Cr, S formed by PBEsol-mB.

To find the origin of ferromagnetism, the total density of state (TDOS) as well as partial density of state (PDOS) for ZnX_2_Se_4_ (X = Ti, V, Cr) chalcogenides are shown in Figure 4a–c. The TDOS exhibit the metallic nature in spin-up channel and semiconductor in spin-down channel. The above discussion shows that transition metals (Ti, V, Cr) doped Zn based chalcogenides have *p-d* exchange interaction with the valance band of host semiconductor ZnX_2_Se_4_ near the Fermi energy (E_F_). Whenever Fermi level is controlled by impurity band gap, the p-type carrier produce ferromagnetism. The impurity explains huge exchange splitting for Ti which decrease from Ti to Cr in continues manner due to increasing number of electrons. According to the crystal field theory, the tetrahedral shape of surrounded anions produce a field, which splits the 3*d* states of (Ti, V, Cr) into *t_2g_(d_Xy_,d_yz_, d_zx_)* states called bonding and *e_g_(d_x2-y2,_d_z2_)* called antibonding states [27,28]. As e_g_ states have low energy in contrast with t_2g_ states, therefore, the energy difference between e_g_ and t_2g_ in terms of crystal field energy is E_cry_ = E_t2g_ − E_eg_. The large direct interchange splitting energy *∆_x_(d)* of 3*d* states for the transition metals (X = Ti, V, Cr) surpasses crystal field energy *(∆_x_(d) > E_cry_)*, which induced ferromagnetism as shown in Table 2. Furthermore, *d*-states of Cr and V atoms are observed to exist at valance band maxima. For Cr doped ZnX_2_Se_4_ alloys, the bonding state e_g_ do not contribute in hybridization process due to six unpaired electrons. While Ti has two unpaired electrons, V has three unpaired electrons and Cr has four unpaired electrons according to electronic configurations.

Moreover, the interchange splitting energy ∆_x_(*pd*) is calculated through the positions of anions or from band structures whose negative value suggest that the down-spin channel is much attractive as compared to the spin-up channel. Further, *p*-*d* repulsive model described the interaction between transition metals 3*d* state and anion p state that keep them away from fermi level because of large splitting into spin-down channel [29,30].

Furthermore, in accordance with the Pauli Exclusion Principle, the unpaired electrons in 3*d* states of transition metal impurity are equal to their magnetic moment. Thus, Ti, V, Cr have two, three and four unpaired electrons in 3*d* states. Therefore, Ti, V, and Cr containing spinels should have magnetic moment 2, 3 and 4 μ_B_. Our computed values of magnetic moments for Ti, V, and Cr containing compounds are 2, 3 and 4 μ_B_ which are consistent with Pauli Exclusion Principle and show the accuracy of our computed results (See Table 3) [31,32]. The Cr has maximum value of magnetic moment and Ti has least value in studied transition metal doped semiconductors. Additionally, the magnetism in transition metal doped semiconductors follows the Hund’s rule, which explains the increase in magnetic moments because of the interaction among *s*, p and d states. The transition metal impurities (Ti, V, Cr) induce magnetic moments induced at Zn and X, Se sites of host semiconductors. Moreover, knowledge of changes at valance edge is essential to find the origin of ferromagnetism. The mean field theory describes the band variation in terms of Hamiltonian, such as H = N_0_ β . Here, N_0_ is cation contribution and β is the *p*-*d* interchange integral. The Se behaves as hole carrier spin and X is impurity spin. This Hamiltonian is dependent on band disturbance that is calculated through the Kondo relations and can be defined in terms of interchange constants N_0_ α and N_0_ β that are calculated from conduction band edge splitting as well as valance band edge splitting by using the following relation [33,34,35,36]:

(5) $${\;N}_{O}\alpha =\frac{{\Delta E}_{C}}{x\langle S\rangle }\;\mathrm{and}\;N_{O}\beta =\frac{{\Delta E}_{V}}{x\langle S\rangle }$$

where, x is the concentration of X ions and 〈S〉 represents the mean value of the magnetic moment. It is obvious that, *s-d* as well as *p-d* mixture are ferromagnetic for the half metallic compounds (Ti, V, Cr). In addition, the more negative value of N_o_ β shows the ferromagnetism in transition metal doped (X = Ti, V, Cr) Zn based alloys is deceases the energy of the system to stabilize ferromagnetism (See Table 2).

The Pauli exclusion principle states that a single quantum state cannot be filled with two identical fermions. Depending upon the number of unpaired electrons in the outermost shell, the magnetic moment of Ti is 2µ_B_, V is 3µ_B_ and Cr is 4µ_B_. This increase in magnetic moment is due to increase in unpaired electrons in the outer shells whose integral values ensure the complete spin polarization (See Table 3) [37,38,39,40].

### 3.3. Transport Properties

**Figure 5:**
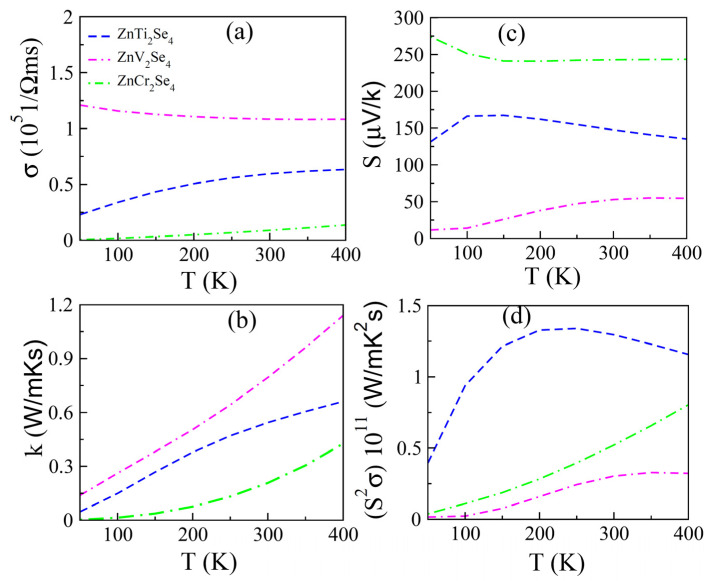
(**a**) *σ*, (**b**) *k*, (**c**) *S* and (**d**) *σ S^2^* of ZnX_2_Se_4_ (X = Ti, V, Cr) computed by Boltz TraP code.

The thermoelectric measurement converts the directly heat energy into electrical energy by thermoelectric generators. Investigation of transport properties usually involve the measurement of electrical conductivity (σ), thermal conductivity (*κ*), Seebeck coefficient (*S*), power factor (PF) and figure of merit (ZT), which quantify the response of materials towards thermoelectric applications [41,42,43,44,45]. Here we have computed these properties using BoltzTraP code. The thermal conductivity has two parts, i.e., electron (*k_el_*) and phonon (*k_ph_*). Here we incorporated the electronic part only because this code could not work for phonon calculations. In addition, this code works well for metals having an excess number of electrons which suppress the lattice contribution in thermal conductivity. The density functional theory perturbation (DFTP) approach is considered more effective to incorporating the phonon part in conductivity, hence the reason for only reporting the electronic part. Figure 5a shows the variation of σ in the temperature range of 0–400 K. For ZnTi_2_Se_4_, it slightly increases in the lower T range (T < 350 K) and become almost constant when temperature increases from 350 K. The ZnV_2_Se_4_ has maximum value of σ which is attributed to the odd number of electrons present in 3*d* states of V [46,47,48,49,50]. The ZnCr_2_Se_4_ has a least value of σ which linearly increased with temperature.

Seebeck coefficient (S) measures the potential gradient developed across two dissimilar materials when these materials meet each other. The variation in S with temperature is shown in Figure 5c in the temperature range 0–400 K. For all compositions, S is positive which reveal the fact that in these compositions, the majority charge carries are holes. The S is maximum for Cr and minimum for V containing compositions. At 400 K, S is 130 µV/K for ZnTi_2_Se_4_, 57 µV/K for ZnV_2_Se_4_ and 245 µV/K for ZnCr_2_Se_4_. The product of σ and S^2^ determine another important factor called power factor (PF), which verify the thermoelectric strength of the material [51]. Figure 5d shows how the power factor varies in the temperature range 0–400 K. The PF is maximum for ZnTi_2_Se_4_ and minimum for ZnV_2_Se_4_. Based on this observation, we can claim that ZnTi_2_Se_4_ is the most suitable candidate among the present compounds for thermoelectric applications.

## 4. Conclusions

In summary, half metallic ferromagnetism and transport behavior of ZnX_2_Se_4_ (X = T, V, Cr) have been comprehensively analyzed. The more energy released in ferromagnetic state than in anti-ferromagnetic state confirms their stability in FM states, which is further ensured by negative formation energy and positive values of cohesive energy. The Curie temperatures of Ti, V and Cr based chalcogenides are 250, 290 and 305 K. The integer magnetic moment and band structures show the 100% spin polarization. The ferromagnetism is explored by strong hybridization, and greater values of exchange energies than crystal field energies. Moreover, negative values of *N_o_β* and pd coupling energy lower the energy of the spin-orbit coupling system to stabilize ferromagnetism and ensures ferromagnetism due to exchange of electrons rather than clustering of magnetic ions. Thermoelectric behavior shows the ZnTi_2_Se_4_ has large value of power factor, which increases its thermoelectric performance.

## Figures and Tables

**Table 1:** The computed a(Å): Lattice constant, B(GPa): Bulk modulus, *E_coh_*: Cohesive energy, ΔH_f_ (eV): Formation energy and T_c_ (K): Curie temperature of ZnX_2_Se_4_ (X = Ti, V, Cr).

Composition	a (Å)	B (GPa)	*E_coh_*	ΔH_f_ (eV)	T_c_ (K)
ZnX_2_Se_4_					
X = Ti	10.69	69.81	4.20	−1.69	250
X = V	10.61	75.48	3.18	−1.32	290
X = Cr	10.57, 10.48 [24]	78.40	2.70	−1.21	305

**Table 2:** The computed (crystal field energy) ΔE_crystal_, interchange energy Δ_x_(*d*), *pd*-exchange energy Δ_x_(*pd*) and interchange constants (*N_o_α* and *N_o_β*) of ZnX_2_Se_4_ (X = Ti, V, Cr).

Composition	(ΔE_crystal_) eV	Δ_x_(*d*) eV	Δ_x_(*pd*) eV	*N_o_α*	*N_o_β*
ZnX_2_Se_4_					
X = Ti	1.44	2.51	−0.338	1.041	−1.069
X = V	2.62	3.50	−0.195	0.182	0.210
X = Cr	1.69	2.08	−0.379	0.531	−0.306

**Table 3:** The computed magnetic moment of whole composition and at Zn, X = Ti, V, Cr and Se sites of ZnX_2_Se_4_ (X = Ti, V, Cr).

Compound	Magnetic Moments (in Terms of Bohr Magnetron, *µ_B_*)			
	Total	Zn-Site	X-Site	S/Se-Site
ZnX_2_Se_4_				
X = Ti	2.000	0.017	0.875	−0.015
X = V	3.000	0.008	1.964	−0.068
X = Cr	4.000, 5.47 [24]	0.004	2.970	−0.057

## Data Availability

Data will be available on request.
