# Correction: Al-Qhtani et al. Half Metallic Ferromagnetism and Transport Properties of Zinc Chalcogenides ZnX_2_Se_4_ (X = Ti, V, Cr) for Spintronic Applications. *Materials* 2022, *15*, 55

**DOI:** 10.3390/ma15072521

**Published:** 2022-03-30

**Authors:** Mohsen Al-Qhtani, Ghulam M. Mustafa, Nasheeta Mazhar, Sonia Bouzgarrou, Qasim Mahmood, Abeer Mera, Zaki I. Zaki, Nasser Y. Mostafa, Saad H. Alotaibi, Mohammed A. Amin

**Affiliations:** 1 Materials Science and Engineering Group, Department of Chemistry, Faculty of Science, Taif University, Taif 21944, Saudi Arabia; mohsen@tu.edu.sa (M.A.-Q.); zakimohamed2000@yahoo.com (Z.I.Z.); nmost69@yahoo.com (N.Y.M.); s.alosaimi@tu.edu.sa (S.H.A.); mohamed@tu.edu.sa (M.A.A.); 2 Department of Physics, Division of Science & Technology, University of Education, Lahore 54000, Pakistan; 3 Department of Physics, University of Lahore, Lahore 05422, Pakistan; nasheen@gmail.com; 4 Laboratoire de Microélectronique et Instrumentation (UR 03/13–04), Faculté des Sciences de Monastir, Avenue de l’Environnement, Monastir 5000, Tunisia; b_sona3@yahoo.fr; 5 Department of Physics, College of Science, Qassim University, P.O. Box 64, Buraydah 51452, Saudi Arabia; 6 Basic and Applied Scientific Research Center, Imam Abdulrahman Bin Faisal University, P.O. Box 1982, Dammam 31441, Saudi Arabia; 7 Department of Physics, College of Science, Imam Abdulrahman Bin Faisal University, P.O. Box 1982, Dammam 31441, Saudi Arabia; 8 Department of Physics, College of Arts and Science, Prince Sattam Bin Abdulaziz University, Wadi Addawasir 11991, Saudi Arabia; abeer.mera047@gmail.com; 9 Department of Physics, Faculty of Science, Kafrelsheikh University, Kafrelsheikh 33516, Egypt

In the original publication [1], Affiliation 10 is not the author’s address in the preparation stage of this article, so it needs to be deleted and listed in the front note: current address. Only the affiliation number has changed: Nasser Y. Mostafa ^1,10^ changed to Nasser Y. Mostafa ^1^.

The authors apologize for any inconvenience caused and state that the scientific conclusions are unaffected. The original article has been updated.
